# Anwendbarkeit von QUIKS bei stationär konservativ behandelten Tumorpatienten

**DOI:** 10.1007/s00482-021-00599-6

**Published:** 2021-10-27

**Authors:** Carmen Roch, Theresa Kress, Joachim Erlenwein, Winfried Meissner, Elmar Marc Brede, Birgitt van Oorschot

**Affiliations:** 1 Interdisziplinäres Zentrum Palliativmedizin, Universitätsklinikum Würzburg, Josef-Schneider-Str. 11, 97080 Würzburg, Deutschland; 2 Klinik und Poliklinik für Anästhesiologie, Intensivmedizin, Notfallmedizin und Schmerztherapie, Universitätsklinikum Würzburg, Josef-Schneider-Str. 2, 97080 Würzburg, Deutschland; 3 Klinik für Anästhesiologie, Universitätsmedizin Göttingen, Göttingen, Deutschland; 4 Klinik für Anästhesie und Intensivmedizin, Universitätsklinikum Jena, Jena, Deutschland; 5 Klinik für Innere Medizin II, Abteilung Palliativmedizin, Universitätsklinikum Jena, Jena, Deutschland

**Keywords:** Schmerzmanagement, Qualitätssicherung, Benchmarking, Tumorschmerz, Konservative Behandlung, Pain management, Quality assurance, Benchmarking, Tumor pain, Conservative treatment

## Abstract

**Hintergrund:**

„Qualitätsverbesserung im konservativen Schmerzmanagement“ (QUIKS), das Modul für nichtoperative Patienten welches an das „QUIPS“-Projekt angelehnt ist, wurde an einer Kohorte Tumorpatienten auf Anwendbarkeit getestet.

**Material und Methoden:**

Prospektiv wurden stationär konservativ behandelte Patienten am Universitätsklinikum Würzburg (UKW) anhand des Ergebnisfragebogens QUIKS zur Qualität der Schmerztherapie befragt (AZ 129/17, Ethikkommission am UKW). Informationen zur Therapie und Demografie wurden dem klinikinternen Dokumentationssystem entnommen.

**Ergebnisse:**

Im Erfassungszeitraum konnten 100 Tumorpatienten aus verschiedenen Kliniken eingeschlossen werden. 74 % der Patienten benötigten Unterstützung bei der Beantwortung des Fragebogens. Funktionelle Einschränkungen oder schmerztherapiebedingte Nebenwirkungen lagen bei 77 % der Patienten vor, im Durchschnitt lagen Schmerzen von 6 auf der numerischen Rating-Skala vor. Die am häufigsten benannten Schmerzentitäten waren Rücken- und Kopfschmerzen. 18 % der Patienten erhielten eine Schmerztherapie mit Opioiden, 26 % mit Nichtopioiden, eine Anpassung der Schmerztherapie erfolgte in 5 % mit Opioiden und in 44 % mit Nichtopioiden, ein Einbezug schmerzmedizinischer Spezialisten erfolgte in 9 %.

**Fazit:**

Die Anwendung des Fragebogens wurde von den Patienten gut akzeptiert, war jedoch mit einem großen Maß an Unterstützung beim Ausfüllen verbunden. Es zeigte sich ein hohes Schmerzniveau während des Krankenhausaufenthalts. Eine Anpassung der Schmerztherapie oder ein Einbezug schmerzmedizinischer Spezialisten erfolgte selten. Die Interpretation bzgl. Aussagen zur Qualität des Schmerzmanagements könnte eingeschränkt sein, da andere (vorbestehende) Schmerzentitäten, wie nichttumorassoziierter Schmerz oder chronischer Tumorschmerz, nicht eindeutig abgrenzbar sind.

**Zusatzmaterial online:**

Die Online-Version dieses Beitrags (10.1007/s00482-021-00599-6) enthält die QUIKS-Prozessparameter und den QUIKS-Ergebnisfragebogen.

## Abstract (en)

**Background:**

Quality improvement in conservative pain management (QUIKS), a module for nonoperative patients in the QUIPS project was tested on a cohort of tumor patients regarding its applicability.

**Material and methods:**

Conservatively treated inpatients at the University Hospital of Würzburg (UKW) were prospectively surveyed on the quality of pain management using the QUIKS outcome questionnaire (AZ 129/17, Ethics Committee at UKW). Information on therapy and demographics was taken from the hospital’s internal documentation system.

**Results:**

During the data collection period 100 conservatively treated inhouse tumor patients from different hospitals were included. Of the patients 74% required assistance in answering the questionnaire. Functional limitations or pain treatment-related side effects were present in 77% of the patients; the average pain level was 6 on the numerical rating scale. The most commonly reported type of pain was back pain and headache. Of the patients 18% received pain therapy with opioids and 26% with nonopioids, adjustment was made in 5% with opioids and in 44% with nonopioids and pain medicine specialists were consulted in 9% of cases.

**Conclusion:**

The application of the questionnaire was well accepted by the patients but required a high level of assistance in completing it. A high level of pain was observed during the hospital stay and the adjustment of pain therapy or the involvement of pain medicine specialists was rare. The interpretation of statements regarding the quality of tumor pain may be limited as other (pre-existing) pain entities, such as nontumor-associated pain or chronic tumor pain could not be clearly delineated.

## Hintergrund

Eine effektive Schmerztherapie gehört zu den wesentlichen Qualitätsmerkmalen stationärer Krankenversorgung [1, 2]. Dennoch zeigen sich immer wieder Versorgungsdefizite bzgl. der Schmerztherapie sowohl in operativen als auch nichtoperativen Fachbereichen in Krankenhäusern [3, 4]. Für Deutschland zeigte sich dies zuletzt in einer vergleichenden Analyse von Daten einer Kohorte von 21.114 operativen Patienten aus 138 deutschen Kliniken [5]. Anhand dreier ausgewählter Outcomeparameter („stärkster berichteter Schmerz“, „schmerzbedingte Einschränkungen bei Belastung“ und „Zufriedenheit mit der Schmerztherapie“) stellte sich insbesondere für Universitätskliniken ein Optimierungsbedarf dar. Insbesondere die Versorgungslage von Tumorschmerzpatienten ist häufig dürftig [6].

Der Goldstandard für die Qualitätssicherung der Schmerztherapie sind patientenberichtete Outcomeparameter („patient-reported outcomes“ [PRO]). Dementsprechend sollte sowohl die Einschätzung der Schmerzintensität als auch die Beurteilung der Qualität der Schmerztherapie nach Möglichkeit immer durch die Patienten selbst erfolgen und nicht durch Pflegende oder andere Professionen fremdeingeschätzt werden [7, 8].

Vor diesem Hintergrund ist die Forderung nach regelmäßiger Qualitätssicherung der Schmerztherapie im Krankenhaus sehr zu unterstützen [1, 9]. Obwohl es klinische Empfehlungen für die Behandlung chronischer Tumorschmerzpatienten gibt [10], fehlen bislang standardisierte und praktikable Konzepte zur Qualitätssicherung der Schmerztherapie für den klinischen Praxisalltag, die sich speziell an patientenberichteten Outcomeparametern orientieren.

Zur Qualitätssicherung der Schmerztherapie in der perioperativen Medizin wurde im Rahmen des Benchmarkingprojekts „Qualitätsverbesserung in der postoperativen Schmerztherapie“ (QUIPS [11]) ein standardisiertes Erhebungsverfahren entwickelt, welches die Erfassung von PRO und Prozessparametern berücksichtigt. Aus diesem ist inzwischen das weltweit größte Akutschmerzregister für die postoperative Schmerztherapie entstanden. Zudem erfolgt die Erfassung von Prozessparametern. Seit Kurzem steht für konservativ behandelte Patienten ein an QUIPS angelehntes Qualitätssicherungsinstrument zur Verfügung („Qualitätsverbesserung im konservativen Schmerzmanagement“ [QUIKS]; [12]).

### Fragestellung

In Vorbereitung eines klinikinternen Qualitätssicherungssystems wurde in einem monozentrischen Projekt der Schmerzambulanz und der Palliativmedizin des Universitätsklinikums Würzburg die Umsetzbarkeit der Qualitätssicherung mit QUIKS erstmals im stationären Kontext gezielt bei Tumorpatienten untersucht.

### Studiendesign und Untersuchungsmethoden

Nach positivem Ethikvotum (AZ 129/17, Ethikkommission am Universitätsklinikum Würzburg [UKW]) wurden vom 28. August 2017 bis zum 1. Dezember 2017 in sechs Kliniken des UKW Tumorpatienten konsekutiv rekrutiert und prospektiv einmalig befragt: Frauenklinik und Poliklinik; Klinik und Poliklinik für Hals‑, Nasen- und Ohrenkrankheiten, plastische und ästhetische Operationen (HNO); Neurochirurgische Klinik und Poliklinik; Klinik und Poliklinik für Strahlentherapie; Klinik und Poliklinik für Urologie und Kinderurologie und Klinik und Poliklinik für Mund‑, Kiefer und plastische Gesichtschirurgie (MKG). Alle volljährigen Tumorpatienten, bei denen während des Aufenthalts auf der Station keine chirurgische Intervention durchgeführt wurde, erhielten nach entsprechender Aufklärung und Einwilligung in die Studienteilnahme am 3. Tag nach der stationären Aufnahme den QUIKS-Fragebogen. Bei Bedarf wurden die Patienten von einer geschulten Mitarbeiterin beim Ausfüllen unterstützt. Diese gehörte nicht zum Behandlungsteam und arbeitete von diesem unabhängig.

### Parametererfassung mit QUIKS

Der QUIKS-Fragebogen (s. Zusatzmaterial online) enthält neben Fragen zu vorbestehenden Schmerzen auch Fragen zu den während des stationären Aufenthalts vorliegenden Schmerzentitäten und Fragen zur Einschätzung der Schmerzintensität anhand der numerischen Rating-Skalen (NRS, 0 = kein Schmerz bis 10 = stärkster vorstellbarer Schmerz) und dichotom (ja/nein) zu Funktion und Therapienebenwirkungen:

- Schmerzen bei Aufnahme (QUIKS 2b)
- Stärkster Schmerz bei Prozeduren/Maßnahmen im Krankenhaus (QUIKS 2c)
- Schmerz in Ruhe in den letzten 24 h (QUIKS 3a)
- Schmerzen bei Belastung in den letzten 24 h (QUIKS 3b)
- Stärkster Schmerz innerhalb der letzten 24 h (QUIKS 3c)
- Beeinträchtigung von Bewegung, Husten oder tiefem Luftholen, Schlafen und Stimmung durch Schmerzen (QUIKS 4a–d)
- Therapiespezifische Nebenwirkungen „Müdigkeit“, „Übelkeit“, „Schwindel“ und „Verstopfung“ (QUIKS 5a–d)

Außerdem wird mit dem QUIKS-Fragebogen erfasst, ob Patienten über die Möglichkeiten der Schmerztherapie informiert wurden (ja/nein, QUIKS 6) und sich gewünscht hätten, mehr Medikamente gegen Schmerzen zu erhalten (ja/nein, QUIKS 7). Wenn Patienten Schmerzen hatten bzw. eine Schmerztherapie durchgeführt wurde, erfolgte zudem die Erfassung der diesbezüglichen Zufriedenheit anhand der NRS (0 = völlig unzufrieden bis 10 = sehr zufrieden).

Als Prozess- und demografische Parameter wurden mit QUIKS die Grund- bzw. Vorerkrankungen, die Schmerzmedikation vor Krankenhausaufnahme und in den letzten 24 h vor der Befragung, individuelle Therapieanordnungen und spezielle Analgesieverfahren sowie die Schmerzdokumentation (ja/nein) erfasst.

Über den QUIKS-Fragebogen hinaus wurde zusätzlich erfasst, wie häufig Palliativmediziner und/oder Schmerztherapeuten in die Behandlung von Tumorschmerz bei diesen Patienten einbezogen wurden.

Die Informationen zur Soziodemografie wurden vom Patienten beantwortet, krankheitsbezogene Daten wurden dem Krankenhausinformationssystem (SAP®, SAP SE, Walldorf, Deutschland) entnommen. Die verschiedenen Tumorentitäten wurden entsprechend den durch die Deutsche Krankenhaus Gesellschaft zertifizierten Organzentren zusammengefasst.

### Statistische Auswertung

Die Auswertung aller erhobenen Daten erfolgte mit SPSS (Statistical Package for Social Science, IBM®), Version 26.

Zunächst erfolgte eine deskriptive Darstellung der Soziodemografie sowie der Ergebnis- und Prozessparameter des QUIKS-Fragebogens. Kontinuierliche Merkmale wurden mit Mittelwert ± Standardabweichung dargestellt. Ordinal skalierte Angaben zur Schmerzintensität und Zufriedenheit auf der NRS wurden anhand des Medians und des 1. und 3. Quartils (Median [1.–3. Quartil]) angegeben.

Die Auswertung bzgl. Schmerzintensität erfolgte an der Gruppe Patienten, die während des stationären Aufenthalts Schmerzen hatten (Fragen 2 und 3: NRS ≥ 1). Bei der Auswertung der Zufriedenheit mit der Schmerztherapie und des Wunschs nach mehr Schmerzmitteln wurde die Gruppe der Patienten mit Schmerzen im Krankenhaus mit der gesamten Stichprobe verglichen. Ob eine Korrelation zwischen Informiertheit der Patienten über die Möglichkeiten einer Schmerztherapie und Zufriedenheit mit der Schmerztherapie bestand, wurde anhand einer biserialen Korrelation überprüft (Signifikanz *p* < 0,05).

## Ergebnisse

### Einschlüsse und Grundcharakteristika

Insgesamt wurden im Erfassungszeitraum 133 Patienten für die Studie als geeignet identifiziert. Zehn Patienten wurden wegen Verständigungsproblemen, Stationswechsel oder Entlassung nicht angefragt und 23 Patienten lehnten eine Studienteilnahme ab (Beteiligungsrate 78,1 %), sodass insgesamt 100 Patienten aus sechs verschiedenen Kliniken eingeschlossen werden konnten (*n* = 30 Patienten aus der Strahlentherapie, *n* = 18 aus der Gynäkologie, *n* = 5 aus der Urologie, *n* = 28 aus der HNO, *n* = 10 aus der Neurochirurgie, *n* = 9 aus der MKG). Bei 59 Patienten wurde die Tumorerkrankung mit kurativer Intention therapiert, 40 Patienten befanden sich in einer Palliativsituation, bei einem Patienten wurde das Staging auf eigenen Wunsch vor Diagnosefindung abgebrochen. 26 Befragte beantworteten die Bögen eigenständig oder mit familiärer Unterstützung, bei 74 Patienten erfolgte dies per Interview (74,0 %). Hierbei benötigten 85,0 % der palliativen Patienten und 66,1 % der kurativen Patienten Unterstützung. Tab. 1 fasst die soziodemografischen und tumorbezogenen Charakteristika zusammen.

**Table 1:** 

	Alle (*n* = 100)
Alter in Jahren, MW ± SD	61,3 ± 12,8
*Geschlecht (%)*	
Männlich	47,0
Weiblich	53,0
*Tumorentität (%)*	
Kopf-Hals-Tumoren	33,0
Gynäkologische Tumoren	21,0
Tumoren des Gastrointestinaltrakts	10,0
Neuroonkologische Tumoren	9,0
Bronchialtumoren	9,0
Urogenitale Tumoren	7,0
Sonstige	11,0

*n* Anzahl, *MW* Mittelwert, *SD* Standardabweichung

### QUIKS-Ergebnisparameter

**Figure 1:**
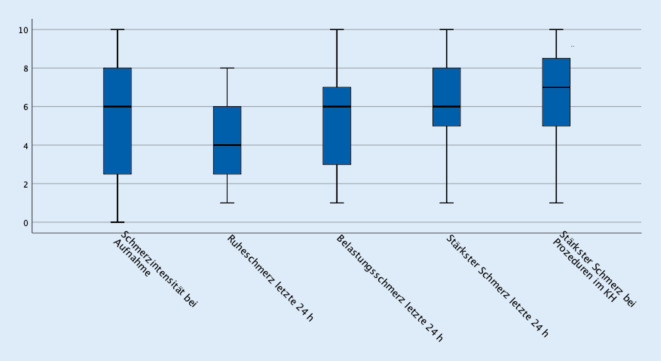

Wenn nicht anders genannt, gab es im Folgenden bei den Ergebnisparametern keine Fehlwerte. 35 % der Befragten berichteten über vorbestehende Schmerzen mit höherer Schmerzintensität (Schmerzintensität „meistens“ NRS ≥ 4), die länger als 3 Monate vor Krankenhausaufnahme bestanden (s. Abb. 1). Bei 68,6 % dieser Patienten war die Schmerzsituation auch der Aufnahmegrund.

**Figure 2:**
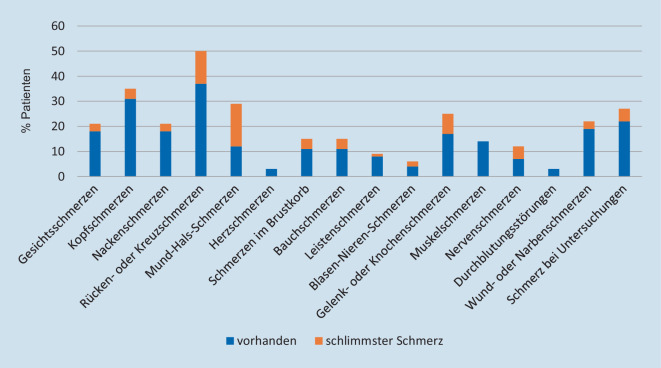

Die häufigsten Schmerzentitäten sowie die am schlimmsten empfundenen Schmerzen sind Abb. 2 zu entnehmen.

Bei der Frage am 3. Tag des stationären Aufenthalts nach stärksten Schmerzen in den letzten 24 h berichteten 67 % aller Patienten über Schmerzen (≥ 1 auf der NRS). Ruhe- bzw. Belastungsschmerzen in den letzten 24 h wurden von 47 % bzw. 52 % der Patienten angegeben. Am Aufnahmetag berichteten 52 % der Patienten über das Vorliegen von Schmerzen. 55 % der Patienten berichteten von Schmerzen (NRS ≥ 1) während Prozeduren im Krankenhaus (s. Abb. 1).

77 % aller Patienten gaben funktionelle Einschränkungen wegen Schmerzen bzw. schmerztherapiebedingter Nebenwirkungen an. Hiervon berichteten 37 % der Patienten, bei Bewegung eingeschränkt zu sein. Weitere 26 % der Patienten beklagten beim Husten oder tiefen Luftholen Beeinträchtigungen, 28 % beim Schlaf und 36 % bei der Stimmung.

62 % der Patienten berichteten über Müdigkeit und 29 % über Übelkeit. 37 % litten unter Schwindel und 19 % unter Obstipation.

**Figure 3:**
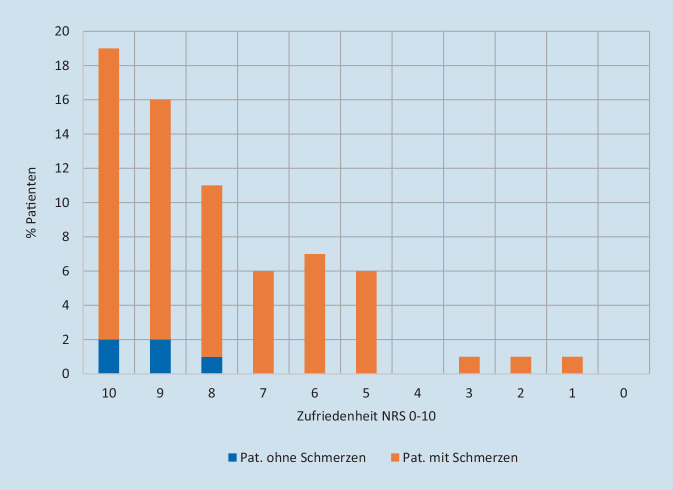

Insgesamt wurde die Zufriedenheit mit der Schmerztherapie von 68 % der Patienten bewertet. Davon gehörten 63 % der Patienten zur Gruppe der Schmerzpatienten. 5 % der Patienten, die die Schmerztherapie bewerteten, gaben jedoch weder bei Aufnahme noch am 3. Tag des Krankenhausaufenthalts Schmerzen an (s. Abb. 3). Die verbleibenden 32 % der Patienten machten keine Angaben zur Zufriedenheit (s. Tab. 2). Zwischen der Informiertheit der Patienten über die Möglichkeiten der Schmerztherapie und der Zufriedenheit mit der Schmerztherapie ließ sich kein Zusammenhang nachweisen (*p* = 0,48; s. Tab. 3).

**Table 2:** 

	Alle *n* = 100	Schmerzpatienten *n* = 76
Wunsch nach mehr Mitteln gegen die Schmerzen (ja)	5,0 %	6,6 %
Wenn Sie Schmerzen hatten bzw. eine Schmerztherapie erhalten haben: Zufriedenheit mit dem Ergebnis der Schmerztherapie (NRS 0–10)	68,0 % 9 (7,0–10,0)	82,9 % 8 (6,0–10,0)

*n* Anzahl

**Table 3:** 

	Alle *n* = 100	Schmerzpatienten *n* = 76
Informiertheit über die Möglichkeiten einer Schmerztherapie (ja)	41,0 %	47,4 %
Schmerzdokumentation in der Kurve (ja)	94,0 %	93,4 %

*n* Anzahl

### QUIKS-Prozess- und demografische Parameter

Bei den Nebendiagnosen waren Herz-Kreislauf-Erkrankungen mit 56 % am häufigsten vertreten, gefolgt von endokrinologischen (33 %) und pulmonalen Erkrankungen. Weitere Nebenerkrankungen lagen im Bereich des Nervensystems (12 %), des Bewegungsapparats (9 %), des Gastrointestinaltrakts (5 %) sowie der Nieren (6 %), der Haut (3 %) und des Blutes (1 %). Bei 27 % der Patienten konnte keine Nebendiagnose erfasst werden.

Bei stationärer Aufnahme waren 26 % der Patienten mit Nichtopioidanalgetika vortherapiert und 18 % mit Opioiden (3,0 % schwach wirksame, 15,0 % stark wirksame). 11 % der Patienten wurden mit Koanalgetika behandelt, 67 % hatten keine regelmäßige Schmerztherapie. Bei Entlassung stieg die Anzahl der Patienten mit Nichtopioidanalgetika auf 44 % und die der Patienten mit schwach wirksamen Opioiden auf 5 %. Weitere schmerztherapeutische Anpassungen konnten nicht festgestellt werden.

### Konsiliarische Betreuung

Bei fünf Patienten wurde ein Konsil in der Schmerzambulanz angefordert, bei einem dieser Patienten auch ein Palliativkonsil. Bei vier weiteren Patienten wurde ausschließlich ein Palliativkonsil angefordert.

## Diskussion

In dieser Studie wurde der QUIKS-Fragebogen, der zur Sicherung der Qualität der Schmerztherapie für konservativ behandelte Patienten entwickelt wurde [12], erstmals spezifisch bei einer reinen Kohorte konservativ behandelter Tumorpatienten, deren Versorgungslage oftmals nicht ausreichend ist, im stationären Setting eingesetzt [6]. Die Ergebnisse deuten an, dass ein großer Anteil der befragten Patienten über Schmerzen berichtet, die mit einem durchschnittlichen NRS-Wert von 6 eine erhebliche Intensität aufweisen. Dieser Wert übersteigt die Schmerzintensität, die von Patienten am 1. postoperativen Tag nach Cholezystektomien oder Gelenkoperationen angegeben wird [5].

Hinsichtlich der Anwendbarkeit zeigte sich eine hohe Akzeptanz bei den Patienten, jedoch auch, dass die Mehrheit der Patienten den Fragebogen nicht selbstständig ausfüllen konnte, sondern Unterstützung in Form eines Interviews in Anspruch nahm. Außerdem zeigten sich Schwierigkeiten in der Einschätzung funktioneller Einschränkungen und insbesondere der Nebenwirkungen bzw. Symptome, die einerseits durch die Schmerztherapie verursacht werden können, andererseits aber auch sehr häufig im Rahmen der malignen Grunderkrankungen in dieser Patientengruppe auftreten [13].

### QUIKS als Instrument im Qualitätsmanagement bei Tumorpatienten

Der QUIKS-Fragebogen wurde zur Qualitätssicherung der Schmerztherapie bei konservativ behandelten Patienten im Krankenhaus entwickelt [12]. Hierunter sind sowohl Patienten mit Tumoren wie auch Patienten mit chronischen oder voranschreitenden Nichttumorerkrankungen erfasst. Im QUIKS-Fragebogen erfolgt eine vollumfängliche Erhebung der bestehenden Schmerzsymptomatik, unabhängig von der Kausalität des Schmerzes und nur bedingt mit dem Zusammenhang der aktuellen Erkrankung. Die Interpretation bzgl. Aussagen zur Qualität des Tumorschmerzes könnte eingeschränkt sein, da andere, vorbestehende oder chronische, Schmerzentitäten nicht eindeutig abgrenzbar sind, wie der große Anteil an vorhandenen Rückenschmerzen exemplarisch zeigt.

Vor einer breiten Einführung zur Qualitätssicherung sollte insbesondere für Tumorpatienten die Grundgesamtheit für die Anwendung von QUIKS möglichst genau definiert werden. Therapieinduzierte schmerzhafte Nebenwirkungen lassen sich oft schwer von Symptomen unterscheiden, die durch die Primärerkrankung oder bestehende Komorbiditäten hervorgerufen sind. So berichten in unserem Kollektiv 63 % der Patienten über bereits bekannte Komorbiditäten. Faktoren, die eine Chronifizierung von Schmerz beim Tumorpatienten begünstigen, werden nicht erfasst [14], was die Interpretation und Bewertung der Qualität der Schmerztherapie bzgl. der Symptome und Nebenwirkungen einschränkt. Diese können im Kontext sowohl der Therapie als auch der Grunderkrankung(en) stehen und sind ein Teilaspekt der schlechteren Versorgungslage von Tumorpatienten [14]. Somit lassen sich hiermit keine Aussagen ausschließlich über die Schmerztherapie machen, sondern eher eine allgemeine Aussage hinsichtlich Symptomkontrolle bzw. Symptomlast. Wegen der unterschiedlichen Behandelbarkeit sollten diese auch bei der Qualitätssicherung bzw. im Benchmarking bei Tumorpatienten als mehrdimensionaler Parameter Berücksichtigung finden, um systematischen Verzerrungen bzw. Missinterpretationen vorzubeugen.

### Zufriedenheit mit der Schmerztherapie

Die befragten Patienten waren überwiegend sehr zufrieden mit der Schmerztherapie. Wie sich bereits bei der klinischen Validierung des QUIKS-Moduls feststellen ließ, ist bei konservativ betreuten Patienten nicht zwangsläufig mit Schmerz bzw. einem vorausgegangenen Schmerzereignis im Rahmen einer medizinischen Prozedur, wie bei einer Operation, zu rechnen [12]. Das zeigte sich auch in der vorliegenden Kohorte. Auch hier gab es Patienten, die „keine Schmerzen“ angaben, jedoch nicht aufgrund einer effektiven Schmerzbehandlung, sondern aufgrund eines fehlenden Schmerzreizes, weder durch Prozeduren noch durch die Erkrankung. Dies führt auch bei unserer Kohorte vermutlich in der Gesamtheit zu Verzerrungen und zu einer zu positiven Bewertung der Qualität der Schmerztherapie.

Auch wenn die Fragebogenstruktur nicht Teil der vorliegenden Erhebung war, sollte diskutiert werden, ob die Frage nach der erhaltenen Schmerztherapie bzw. empfundenen Schmerzen im Fragebogen nicht ggf. besser zu Beginn gestellt werden sollte. Damit würde die Beurteilung der Schmerztherapie nur dann angeboten, wenn die Frage nach Schmerzen oder der erhaltenen Schmerztherapie bejaht wurde.

Bei Analysen mit Daten aus dem operativen Modul QUIPS zeigte sich ein deutlicher Zusammenhang zwischen der Qualität der Schmerztherapie und der Informiertheit der Patienten über die Möglichkeiten der postoperativen Schmerztherapie [5]. Dieser Zusammenhang ließ sich bei den Patienten unserer Kohorte nicht zeigen. Hierbei sollte jedoch auch bedacht werden, dass angesichts der zu erwartenden Schmerzen eine Aufklärung über die Möglichkeiten der postoperativen Schmerztherapie inzwischen etablierter Standard ist und ggf. im konservativ stationären Setting noch weniger etabliert ist. Zudem bestehen sowohl bei operativen als auch bei konservativen Patienten oft vielfach auch andere Schmerzentitäten, die nicht in direktem Zusammenhang mit der aktuellen Behandlung stehen [12].

### Schmerz- und palliativmedizinischer Versorgungsrahmen

Die innerklinische Schmerzbehandlung obliegt den Primärbehandlern der bettenführenden Abteilung. Schmerz- und palliativmedizinisch spezialisierte Dienste behandeln Patienten anderer Abteilungen im Rahmen von Konsilleistungen oder Liaisonbetreuung mit [10]. Gerade im Kontext von Patienten mit Tumorerkrankungen bzw. im Rahmen der schmerz- und palliativmedizinischen Dienste ist Schmerz zwar ein sehr häufiges Symptom, steht aber in einer Reihe mit anderen stark belastenden Symptomen. Neben Symptomkontrolle stehen hinsichtlich der spezialisierten Betreuung nicht nur Krankheitsverarbeitung und Akzeptanz, sondern auch sozialrechtliche und Versorgungsfragen im Vordergrund der Betreuung [10, 15, 16]. Auch bei den im vorliegenden Kollektiv erfassten Tumorpatienten wurden spezialisierte schmerztherapeutische und/oder palliativmedizinische Dienste häufig (noch) nicht involviert, auch wenn sich ein positiver Effekt auf die Lebensqualität und Symptomkontrolle der Patienten vermuten lässt [17]. Wie sich auch in unserer Erhebung zeigen lässt, werden die Möglichkeiten spezialisiert-schmerztherapeutischer oder palliativmedizinischer Ansätze zu selten umgesetzt, obwohl sich bei einem Großteil der erfassten Patienten durchaus eine hohe Symptombelastung darstellen ließ. Eine palliativmedizinische oder schmerztherapeutische Mitbeurteilung erfolgte dennoch lediglich bei 9 % der Patienten. Hinsichtlich solcher Versorgungsfragen wiederum lässt sich QUIKS zur Qualitätssicherung gut einsetzen und vermag hier auch Unterschiede der Versorgungsqualität bzgl. des Versorgungsrahmens aufzuzeigen.

### Möglicher Erkenntnisgewinn durch QUIKS bei der Versorgung von Tumorpatienten

Bereits 1999 konnte de Wit belegen [18], dass die Bewertung der Qualität der Schmerztherapie von dem Instrument abhängt, das zur Identifizierung der Schmerzen genutzt wurde. Aufgrund dieses Wissens sollte die Nutzung von PRO auch in der Qualitätssicherung weiter unterstützt werden. PRO bieten nicht nur die Möglichkeit, Symptome von Patienten verlässlich zu erfassen, sondern auch die Chance, die Kommunikation zwischen Arzt und Patient zu verbessern [19]. Außerdem besteht die Chance, die Anamnese sinnvoll zu ergänzen und eventuelle Lücken zu schließen. Magee et al. berichteten 2018, dass bei Patienten mit Tumorschmerz häufig unterschiedliche interindividuelle Antworten erfasst werden. Da bekannt ist, dass sich die Selbsteinschätzung der Patienten deutlich von den Erhebungen des Krankenhauspersonals unterscheidet [20], dürfen die mit QUIKS erfassten Daten nur bedingt mit Daten, die bereits anamnestisch zur klinischen Symptomerfassung erhoben wurden, direkt verglichen werden, da die Zielsetzung der Erhebung eine andere ist [12]. Die Nutzung ausschließlich unidimensionaler Schmerzassessments verschlechtert jedoch die Erfassung von Schmerzen [21], was sich vermutlich auch auf die Qualitätssicherung auswirken wird. QUIKS bietet für Tumorpatienten eine Möglichkeit der Schmerzerfassung, die sowohl die Schmerzintensität bei unterschiedlichen Aktivitätsniveaus und Behandlungssituationen wie auch die Beeinträchtigung durch den Schmerz erfasst. Somit ist auch in der Qualitätssicherung die Nutzung eines zusätzlichen Tools mit patientenberichteten Symptomen eine Bereicherung, bekräftigt die bereits bestehenden Empfehlungen zur Erhebung von PRO [2] und erweitert deren klinischen Einsatzbereich. Im Hinblick auf Anwendbarkeit und Erkenntnisgewinn durch QUIKS in einem reinen Kollektiv von Tumorpatienten sollte kritisch bedacht werden, dass 74 % der Patienten bei der Befragung Unterstützung benötigten. Diese Unterstützung konnte im Rahmen der Studie durch eine 1:1-Befragung per Interview durch Vorlesen der Fragen und Antwortmöglichkeiten angeboten werden, auch um eine möglichst lückenlose Erhebung zu erzielen. Um auch in der Qualitätssicherung möglichst vollständige Daten zu erfassen, müssten bei der Einführung in die Routine hier gegebenenfalls entsprechend mehr Ressourcen bedacht und bereitgestellt werden.

### Limitationen

Die Anwendbarkeit von QUIKS wurde prospektiv ausschließlich an einem Kollektiv konservativ behandelter Tumorpatienten untersucht. Anhand von QUIKS ist keine Unterscheidung zwischen einem tumorassoziierten Schmerz oder einem nichttumorassoziierten Schmerz möglich. Dabei war das Kollektiv aufgrund der Rekrutierung in bestimmten Kliniken vorselektiert und entspricht vermutlich nicht ganz einem durchschnittlichen Kollektiv an Tumorpatienten. Hinzu kam die Selektion konservativ behandelter Patienten, die wiederum einen relevanten Anteil an Patienten mit Tumorerkrankungen im Krankenhaus im operativen Versorgungsrahmen ausschloss. Weder das Tumorstadium oder das Vorliegen chronischer Tumorschmerzen konnte abgebildet werden, noch die damit möglicherweise verbundenen Chronifizierungsprozesse erfasst werden. Hierbei sollte jedoch bedacht werden, dass bereits van den Beuken et al. berichteten, dass ein fortgeschrittenes Tumorstadium nicht zwingend mit einer großen Schmerzintensität einhergeht, da auch in frühen Erkrankungsstadien große Symptomlast vorliegen kann [6]. Die Ergebnisse und Aussage zur Anwendbarkeit sind somit nicht auf die Gesamtgruppe der schmerztherapeutisch und palliativmedizinisch behandelten Tumorpatienten im Krankenhaus übertragbar.

## Fazit für die Praxis

- Die Befragung mit QUIKS wurde gut angenommen. Der personelle Aufwand ist in der Durchführung der Qualitätssicherung in diesem Kollektiv nicht zu unterschätzen und muss auch vor dem Hintergrund einer vollständigen Datenakquise bedacht werden. Die im Mittel angegebene Schmerzintensität ist ausgesprochen hoch. Vor dem routinemäßigen Einsatz bei Tumorpatienten sollte die Fragebogenstruktur angepasst werden, um deutlicher das Patientenoutcome auf einen Therapieeffekt zu beziehen. Eine Unterscheidung der Schmerzursache als Filterfrage könnte hier hilfreich sein.
- Weiterhin sollte insbesondere in der Gruppe der Tumorpatienten bedacht werden, dass krankheitsspezifische Symptome nur sehr schwer von Therapienebenwirkungen getrennt werden können. Somit wird hier immer die Gesamtlast belastender Symptome beurteilt, ohne diese nach Ursachen differenzieren zu können. Gleiches gilt für die Zufriedenheit, da diese immer die Symptomlinderung im Rahmen der Therapie umfasst und nicht nur der Schmerzsymptomatik. Obwohl viele Patienten in den Ergebnisparametern eine deutliche Symptomlast berichteten, wurden spezialisierte Dienste, die sich ausschließlich auf die Verbesserung der Symptomlast konzentrieren, sehr selten in Anspruch genommen.

## Supplementary Information





