# Supplementary material for: Applicability of QUIKS in conservatively treated tumor inpatients
Source: Schmerz. 2021 Oct 27;36(5):342–9. [Article in German] doi: 10.1007/s00482-021-00599-6 (PMC9512755; doi:10.1007/s00482-021-00599-6)
Supplement: Supplementary file 1 [file 482_2021_599_MOESM1_ESM.pdf]

# QUIKS Prozess-Parameter

## DEMOGRAPHIE

**D1 Nummer:** ____________________ **D5 Station:** ____________________

**D2 Geschlecht:** ❑ männlich ❑ weiblich

**D3 Alter:** ❑ 18-20 ❑ 21-30 ❑ 31-40 ❑ 41-50 ❑ 51-60
❑ 61-70 ❑ 71-80 ❑ 81-90 ❑ 91-100 ❑ über 100 ❑ nicht erhebbar

**D4 Kalenderwoche Aufnahme:** __ __ **im Jahr**: __ __ __ __

**D6 Fachabteilung:**

❑ Innere Medizin ohne Spezialisierung
❑ Kardiologie
❑ Gastroenterologie
❑ Nephrologie
❑ Pulmologie
❑ Rheumatologie
❑ Häm./ Onkologie
❑ Endokrinologie
❑ Neurologie
❑ Psychiatrie
❑ Psychosomatik
❑ Dermatologie
❑ Palliativmedizin
❑ sonstige: ____________________

**D7 Befragungstag *nach* stationärer Aufnahme:**
❑ 1 ❑ 2 **❑ 3** ❑ 4 ❑ 5 ❑ 6 ❑ 7 ❑ nicht erhebbar

**D8 Einschluss des Patienten nicht möglich aus folgendem Grund:**

❑ Patient befindet sich auf einer anderen Station
❑ Patient lehnt die Befragung ab
❑ Patient ist verwirrt/hat Verständnisschwierigkeiten
❑ Patient ist sediert / schläft
falls ja, bitte auswählen:
❑ leicht schläfrig
❑ häufig müde, leicht erweckbar
❑ tief schlafend, schwer erweckbar (Bitte unbedingt Prozessparameter erheben!)
❑ nicht erweckbar (Bitte unbedingt Prozessparameter erheben!)
❑ Sonstige Gründe
❑ Patient ist nicht anwesend / bereits entlassen
❑ Patient hat bereits teilgenommen
❑ Patient spricht kein Deutsch

## FREIFELDER (für eigene Fragestellungen können Sie bis zu 8 Felder nutzen)

| | |
|---|---|
| **F1** | **F5** |
| **F2** | **F6** |
| **F3** | **F7** |
| **F4** | **F8** |

Copyright Winfried Meißner/ Uniklinikum Jena. Nutzung durch Dritte vorbehaltlich Genehmigung.

| | | | | | | | |
|---|---|---|---|---|---|---|---|
| **H1 *<br>Herz** | ❑ Endokarditis (infekt.) | ❑ Endokarditis (rheum.) | ❑ Myokarditis | ❑ Perikarditis | ❑ Herzklappeninsuff. | ❑ Herzklappenstenose | ❑ sonst. Herzvitium |
| | ❑ Herzinsuffizienz | ❑ dekomp. Herzinsuff. | ❑ ACS | ❑ trans. Herzinfarkt | ❑ KHK | ❑ prim. Rhythmusstörung | |
| **H2 *<br>Kreislauf** | ❑ art. Hypertonie | ❑ pulm. Hypertonie | ❑ Synkope | ❑ Thrombose | ❑ Thrombophlebitis | ❑ pAVK (ohne Ulcus, nicht akut) | ❑ per. Embolie |
| | ❑ Lungenembolie | ❑ Aneurysma | ❑ Varikosis | ❑ chron. ven. Insuffizienz | ❑ Lymphödem /Angitis | | |
| **H3 *<br>Magen/Darm** | ❑ Gastritis / Duodenitis | ❑ Gastroenteritis | ❑ Refluxösophagitis | ❑ Sigmadivertikulitis | ❑ Appendizitis | ❑ Colitis Ulcerosa | ❑ M. Crohn |
| **H4<br>Leber/Pankreas/Milz** | ❑ Gallensteine | ❑ Cholezystitis | ❑ akute Pankreatitis | ❑ chron. Pankreatitis | ❑ Hepatitis | | |
| **H5<br>*Stoffwechsel/Hormone** | ❑ Diabetes mellitus Typ 1 | ❑ Diabetes mellitus Typ 2 | ❑ Hypothyreose | ❑ Hyperthyreose | ❑ Erkrankungen der Nebenschilddrüse | ❑ Störungen des Aldosteronsystems | ❑ M. Cushing |
| | ❑ Diabetes insipidus | ❑ Osteoporose | ❑ Porphyrien | ❑ Adipositas | ❑ Hyperurikämie (nicht akut) | ❑ Gicht | |
| **H6 *<br>Niere** | ❑ Nephritis (infekt.) | ❑ Nephritis (nicht infekt.) | ❑ Nierensteine | ❑ Harnwegsinfekt | ❑ Niereninsuffizienz (ohne Dialyse) | ❑ Niereninsuffizienz (mit Dialyse) | ❑ autoimmune Nephropathie |
| **H7 *<br>Lunge** | ❑ Asthma bronchiale | ❑ COPD/ obstruktive Lungenerkrankungen | ❑ Lungenfibrose/ restriktive Lungenerkrankungen | ❑ Pneumonie (bakt.) | ❑ Pneumonie (viral/atypisch) | ❑ Pneumonie (sonst.) | ❑ Pleuritis (ohne Pneumonie) |
| | ❑ Pneumothorax | ❑ Schlafapnoe | | | | | |
| **H8 *<br>Nervensystem** | ❑ TIA | ❑ Schlaganfall | ❑ ICB | ❑ Synkope | ❑ Schwindel | ❑ Epilepsie | ❑ Multiple Sklerose |
| | ❑ M. Parkinson | ❑ Demenz | ❑ Bandscheibenvorfall | ❑ Kopfschmerz | ❑ Meningitis (bakt.) | ❑ Meningitis (viral) | ❑ Meningitis (sonst.) |
| | ❑ Restless legs | ❑ Polyneuropathie | ❑ sonst. Neuropathie | | | | |
| **H9 *<br>Haut** | ❑ Ulcus cruris (art.) | ❑ Ulcus cruris (ven.) | ❑ Ulcus cruris mixtum/ sonstiges Ulcus cruris | ❑ Pyoderma gangrenosum | ❑ Malum perforans | ❑ Dekubitus | ❑ Erysipel/Pyodermie |
| | ❑ Herpes Zoster/ Herpes simplex | ❑ Urtikaria | ❑ Psoriasis | ❑ Ekzem | ❑ Exanthem (inkl. Arznei-, Virusexanthem) | ❑ Kutanes Lymphom (CTCL, CBCL) | ❑ Vaskulitis |
| | ❑ Erythema exsudativum multiforme (EEM), Stevens-Johnson-Syndrom (SJS), Toxisch-epidermale-Nekrolyse (TEN), Staphylococcal scalded skin syndrome (SSSS) | | ❑ Blasenbildende Autoimmundermatose (inkl. Bullöses Pemphigoid, Epidermolysis bullosa acquisita, Pemphigus vulgaris, Pemphigus foliaceus) | | ❑ Lupus erythematodes (inkl. SLE, SCLE, CDLE) | ❑ Sklerodermie | ❑ CREST |
| | ❑ Dermatomyositis | ❑ Graft versus Host Disease (GvHD) | ❑ Acne inversa | | | | |
| **H10 *<br>Bewegungsapparat** | ❑ Rheum. Arthritis | ❑ Psoriasisarthritis | ❑ Arthrose | ❑ Polyarthrose | ❑ Polymyositis | ❑ Prog. syst. Sklerose | ❑ Sjögren-Syndrom |
| **H11 *<br>Infektion** | ❑ HIV | ❑ AIDS | ❑ Influenza | ❑ sonst. Infektion | | | |
| **H12 *<br>Blut** | ❑ Mangelanämie | ❑ Sichelzellanämie | | | | | |
| **H13 *<br>Maligne Neoplasien**<br>*(als Nebendiagnose erfassen, nur wenn aktiv, oder konkreter Verdacht auf Rezidiv !)* | ❑ Gehirn | ❑ Rückenmark | ❑ Akute Leukämie | ❑ Chron. Leukämie | ❑ HNO | ❑ Mund | ❑ Ösophagus |
| | ❑ Magen | ❑ Dünndarm | ❑ Dickdarm (sonst.) | ❑ Rektum | ❑ Leber | ❑ Pankreas | ❑ Haut |
| | ❑ Knochen | ❑ Plasmozytom | ❑ Niere | ❑ Harnleiter/-blase | ❑ Prostata | ❑ Hoden | ❑ Brust |
| | ❑ Uterus | ❑ Ovarien | ❑ sonstiges | | | | |

**Hauptdiagnose in der Liste umkreisen, Nebendiagnosen ankreuzen* **Hauptdiagnose: □ nicht erhebbar □ sonstige Hauptdiagnose** (bitte hier ausschreiben):

Copyright Winfried Meißner/ Uniklinikum Jena. Nutzung durch Dritte vorbehaltlich Genehmigung.

# Analgetische Vormedikation

**Welche systemische Schmerztherapie wurde vor stationärer Aufnahme durchgeführt (feste Vormedikation?**

**Hat der Patient vor seinem Krankenhausaufenthalt Opioide gegen chronische Schmerzen genommen?**

❑ ja ❑ nein ❑ nicht erhebbar

| A1 Nicht-Opioid | ❑ keins gegeben | | ❑ nicht erhebbar | |
|---|---|---|---|---|
| **Applikation** | **p.o.** | **i.v.** | **i.m.** | **supp.** |
| Celecoxib | ❑ mg | | | |
| Etoricoxib | ❑ mg | | | |
| Parecoxib | | ❑ mg | | |
| ASS | ❑ mg | ❑ mg | | |
| Ibuprofen | ❑ mg | | | ❑ mg |
| Diclofenac | ❑ mg | ❑ mg | ❑ mg | ❑ mg |
| Indometacin | ❑ mg | | | ❑ mg |
| Meloxicam | ❑ mg | | | |
| Naproxen | ❑ mg | | | |
| Metamizol | ❑ mg | ❑ mg | ❑ mg | ❑ mg |
| Paracetamol | ❑ mg | ❑ mg | ❑ mg | ❑ mg |

| A2 Opioid | ❑ keins gegeben | | | | ❑ nicht erhebbar | | | |
|---|---|---|---|---|---|---|---|---|
| **Applikation** | **p.o. unret.** | **p.o. retard** | **i.v.** | **i.m.** | **s.c.** | **supp.** | **s.l./muco.** | **transd.** |
| Tilidin/(Naloxon)* | ❑ mg | ❑ mg | | | | | | |
| Tramadol | ❑ mg | ❑ mg | ❑ mg | ❑ mg | ❑ mg | ❑ mg | | |
| Tapentadol | ❑ mg | ❑ mg | | | | | | |
| Morphin | ❑ mg | ❑ mg | ❑ mg | ❑ mg | ❑ mg | ❑ mg | | |
| Hydromorphon | ❑ mg | ❑ mg | ❑ mg | ❑ mg | ❑ mg | | | |
| Oxycodon | ❑ mg | ❑ mg | ❑ mg | ❑ mg | ❑ mg | | | |
| Oxycodon/(Naloxon)* | ❑ mg | ❑ mg | | | | | | |
| Buprenorphin | | | ❑ mg | ❑ mg | ❑ mg | | ❑ mg | ❑ µg/h |
| Fentanyl | ❑ µg | | ❑ µg | ❑ µg | ❑ µg | | ❑ µg | ❑ µg/h |
| Pethidin | ❑ mg | | ❑ mg | ❑ mg | ❑ mg | ❑ mg | | |
| Piritramid | | | ❑ mg | ❑ mg | ❑ mg | | | |

*Dosisangaben beziehen sich auf das Opioid

Copyright Winfried Meißner/ Uniklinikum Jena. Nutzung durch Dritte vorbehaltlich Genehmigung.

| A3 Ko-Analgetika | ❑ keins gegeben | | ❑ nicht erhebbar | | | |
|---|---|---|---|---|---|---|
| **Applikation** | **p.o.** | **retard** | **i.v.** | **i.m.** | **s.c.** | **supp** |
| Pregabalin | ❑ mg | | | | | |
| Gabapentin | ❑ mg | | | | | |
| Carbamazepin | ❑ mg | ❑ mg | | | | |
| Amitryptilin | ❑ mg | ❑ mg | ❑ mg | ❑ mg | ❑ mg | |
| Trimipramin | ❑ mg | | | | | |
| Duloxetin | ❑ mg | | | | | |
| Citalopram | ❑ mg | | ❑ mg | | | |
| Sertralin | ❑ mg | | | | | |
| Doxepin | ❑ mg | | ❑ mg | ❑ mg | | |
| Lorazepam | ❑ mg | | ❑ mg | | | |
| Diazepam | ❑ mg | | ❑ mg | ❑ mg | | ❑ mg |
| Flupirtin | ❑ mg | ❑ mg | ❑ mg | ❑ mg | | ❑ mg |

**andere:**

Copyright Winfried Meißner/ Uniklinikum Jena. Nutzung durch Dritte vorbehaltlich Genehmigung.

## STATION

**Welche systemische Schmerztherapie wurde auf der Station am Vortag (gesamte 24h des vorherigen Tages) durchgeführt?**

| **S1 Nicht-Opioid** | **❑ keins gegeben** | | **❑ nicht erhebbar** | | |
|---|---|---|---|---|---|
| **Applikation** | **p.o.** | **i.v.** | **i.m.** | **supp.** | **entspricht Vormedikation** |
| Celecoxib | ❑ mg | | | | ❑ |
| Etoricoxib | ❑ mg | | | | ❑ |
| Parecoxib | | ❑ mg | | | ❑ |
| ASS | ❑ mg | ❑ mg | | | ❑ |
| Ibuprofen | ❑ mg | | | ❑ mg | ❑ |
| Diclofenac | ❑ mg | ❑ mg | ❑ mg | ❑ mg | ❑ |
| Indometacin | ❑ mg | | | ❑ mg | ❑ |
| Meloxicam | ❑ mg | | | | ❑ |
| Naproxen | ❑ mg | | | | ❑ |
| Metamizol | ❑ mg | ❑ mg | ❑ mg | ❑ mg | ❑ |
| Paracetamol | ❑ mg | ❑ mg | ❑ mg | ❑ mg | ❑ |

| **S2 Opioid** | **❑ keins gegeben** | | | | | **❑ nicht erhebbar** | | | |
|---|---|---|---|---|---|---|---|---|---|
| **Applikation** | **p.o. unret.** | **p.o. retard** | **i.v.** | **i.m.** | **s.c.** | **supp.** | **s.l./muco.** | **transd.** | **entspricht Vormedikation** |
| Tilidin/(Naloxon)* | ❑ mg | ❑ mg | | | | | | | ❑ |
| Tramadol | ❑ mg | ❑ mg | ❑ mg | ❑ mg | ❑ mg | ❑ mg | | | ❑ |
| Tapentadol | ❑ mg | ❑ mg | | | | | | | ❑ |
| Morphin | ❑ mg | ❑ mg | ❑ mg | ❑ mg | ❑ mg | ❑ mg | | | ❑ |
| Hydromorphon | ❑ mg | ❑ mg | ❑ mg | ❑ mg | ❑ mg | | | | ❑ |
| Oxycodon | ❑ mg | ❑ mg | ❑ mg | ❑ mg | ❑ mg | | | | ❑ |
| Oxycodon/(Naloxon)* | ❑ mg | ❑ mg | | | | | | | ❑ |
| Buprenorphin | | | ❑ mg | ❑ mg | ❑ mg | | ❑ mg | ❑ µg/h | ❑ |
| Fentanyl | ❑ µg | | ❑ µg | ❑ µg | ❑ µg | | ❑ µg | ❑ µg/h | ❑ |
| Pethidin | ❑ mg | | ❑ mg | ❑ mg | ❑ mg | ❑ mg | | | ❑ |
| Piritramid | | | ❑ mg | ❑ mg | ❑ mg | | | | ❑ |

*Dosisangaben beziehen sich auf das Opioid

Copyright Winfried Meißner/ Uniklinikum Jena. Nutzung durch Dritte vorbehaltlich Genehmigung.

| S3 Ko-Analgetika | ❑ keins gegeben | | ❑ nicht erhebbar | | | | |
|---|---|---|---|---|---|---|---|
| **Applikation** | **p.o.** | **retard** | **i.v.** | **i.m.** | **supp.** | **s.c.** | **entspricht Vormedikation** |
| Pregabalin | ❑ mg | | | | | | ❑ |
| Gabapentin | ❑ mg | | | | | | ❑ |
| Carbamazepin | ❑ mg | ❑ mg | | | | | ❑ |
| Amitryptilin | ❑ mg | ❑ mg | ❑ mg | ❑ mg | | ❑ mg | ❑ |
| Trimipramin | ❑ mg | | | | | | ❑ |
| Duloxetin | ❑ mg | | | | | | ❑ |
| Citalopram | ❑ mg | | ❑ mg | | | | ❑ |
| Sertralin | ❑ mg | | | | | | ❑ |
| Doxepin | ❑ mg | | ❑ mg | ❑ mg | | | ❑ |
| Lorazepam | ❑ mg | | ❑ mg | | | | ❑ |
| Diazepam | ❑ mg | | ❑ mg | ❑ mg | ❑ mg | | ❑ |
| Flupirtin | ❑ mg | ❑ mg | ❑ mg | ❑ mg | ❑ mg | | ❑ |

**andere:**

**Ist eine Individuelle Therapieanordnung für die Schmerztherapie auf der Station vorhanden?**

| **S4 Therapieanordnung** | ❑ Ja | ❑ Nein |
|---|---|---|
| **Basisanalgesie angeordnet** | ❑ Ja | ❑ Nein |
| **Bedarfsmedikation angeordnet** | ❑ Ja | ❑ Nein |

**Ist eine Schmerzdokumentation (z.B. Kurve) auf der Station erfolgt?**

| **S5 Schmerzdokumentation** | ❑ Ja | ❑ Nein |
|---|---|---|

**Wurde ein spezielles Analgesieverfahren durchgeführt?**

| **S6 Spezielles Analgesieverfahren** | **❑ keine erhalten** | **❑ nicht erhebbar** |
|---|---|---|
| ❑ PCIA (intravenös)<br>❑ PCA (iontophoretisch)<br>❑ PCA sublingual | ❑ PCEA (epidural) | ❑ PCRA (regional) |

Copyright Winfried Meißner/ Uniklinikum Jena. Nutzung durch Dritte vorbehaltlich Genehmigung.
