# Supplementary material for: Applicability of QUIKS in conservatively treated tumor inpatients
Source: Schmerz. 2021 Oct 27;36(5):342–9. [Article in German] doi: 10.1007/s00482-021-00599-6 (PMC9512755; doi:10.1007/s00482-021-00599-6)
Supplement: Supplementary file 2 [file 482_2021_599_MOESM2_ESM.pdf]

Qualitätsverbesserung in der Schmerztherapie konservativer (nicht-operativer) Patienten (QUIKS)

Ein Modul des QUIPS-Projekts zum Benchmarking der Schmerztherapie bei konservativen Patienten

DEMOGRAPHIE

D1 Nummer: ________________
(Hier bitte nur die Nummer eintragen, die für diesen Datensatz bei der webbasierten Eingabe des Fragebogens generiert wird.)

D2 Station: ________________ **D3 Fachrichtung/Klinik:** ________________

Copyright Winfried Meißner/ Uniklinikum Jena. Nutzung durch Dritte vorbehaltlich Genehmigung.

Sehr geehrte Patientin, sehr geehrter Patient,
wir führen an dieser Klinik eine Befragung zu gesundheitlichen Beschwerden im Rahmen stationärer Behandlungen durch. Wir möchten Sie daher höflich bitten, die folgenden Fragen zu beantworten. Die Datenerhebung ist freiwillig. Ihre persönlichen Angaben werden den behandelnden Ärztinnen und Ärzten und dem Pflegepersonal nicht zugänglich gemacht und nach Datenerfassung anonymisiert, d.h. ohne Name oder Geburtsdatum erfasst. Falls Sie sich nicht zu einer Teilnahme entschließen können, hat das keine Auswirkungen auf ihre weitere Behandlung.

Kreuzen Sie bitte auf den Skalen jeweils die Zahl an, die für Ihren Schmerz zutrifft („0“ bedeutet Schmerzfreiheit und „10“ stärkster vorstellbarer Schmerz).

1a. Hatten Sie **ständige Schmerzen**, die **drei Monate oder länger** andauerten, **bevor** Sie ins Krankenhaus gekommen sind?

❑ Ja ❑ Nein

1b. Falls ja, **wie stark** waren diese Schmerzen meistens?

0	1	2	3	4	5	6	7	8	9	10

Kein Schmerz — Stärkster vorstellbarer Schmerz

1c. Falls ja, waren diese Schmerzen Grund für die Krankenhausaufnahme?

❑ ja ❑ nein ❑ weiß nicht

Bei den folgenden Fragen geht es um Ihre Schmerzen, die während des Aufenthalts im Krankenhaus bestanden:

2a. Haben Sie bisher **während Ihres Aufenthaltes** im Krankenhaus an **einer oder mehreren** der folgenden Schmerzarten gelitten?

Bitte kreuzen Sie in der ersten Spalte die Kästchen der Schmerzarten an, die bei Ihnen zutreffen. Geben Sie bitte auch die Schmerzen an, die eventuell in keinem Zusammenhang mit ihrer aktuellen Erkrankung stehen. Dann kreuzen Sie in der zweiten Spalte an, welche der Schmerzarten für Sie am schlimmsten war/ist.	**Ja** *(hier alles ankreuzen, was bei Ihnen zutrifft)*	**Der schlimmste Schmerz** *(hier bitte nur eines ankreuzen!)*
Gesichtsschmerzen	❑	❑
Kopfschmerzen	❑	❑
Nackenschmerzen	❑	❑
Rücken- oder Kreuzschmerzen	❑	❑
Mund-/ Halsschmerzen (z.B. beim Schlucken/Essen)	❑	❑
Herzschmerzen (z.B. Angina pectoris)	❑	❑
Schmerzen im Brustkorb (z.B. beim Husten, Atmen)	❑	❑
Bauchschmerzen	❑	❑
Leistenschmerzen	❑	❑
Blasen- oder Nierenschmerzen	❑	❑
Gelenk- oder Knochenschmerzen	❑	❑
Muskelschmerzen	❑	❑
Nervenschmerzen (z.B. Ischias, Nervenverletzungen oder Polyneuropathie bei Zuckerkrankheit, Gürtelrose)	❑	❑
Schmerzen bei Durchblutungsstörungen	❑	❑
Wund- oder Narbenschmerzen	❑	❑
Schmerz bei Untersuchung, Eingriff, Punktion, Behandlung	❑	❑
Andere Schmerzen (bitte benennen): ___________________	❑	❑

Copyright Winfried Meißner/ Uniklinikum Jena. Nutzung durch Dritte vorbehaltlich Genehmigung.

Kreuzen Sie bitte auf den Skalen jeweils die Zahl an, die für Ihren Schmerz zutrifft („0“ bedeutet Schmerzfreiheit und „10“ stärkster vorstellbarer Schmerz).

2b. Wie stark waren Ihre Schmerzen **bei der Aufnahme** ins Krankenhaus?

0	1	2	3	4	5	6	7	8	9	10

Kein Schmerz — Stärkster vorstellbarer Schmerz

2c. Bitte kreuzen Sie hier den **stärksten Schmerz** an**,** den Sie seit Ihrem Krankenhausaufenthalt bei **schmerzhaften Prozeduren/Maßnahmen** (z.B. Untersuchung, Eingriff, Punktion, Behandlung) hatten.

0	1	2	3	4	5	6	7	8	9	10

Kein Schmerz — Stärkster vorstellbarer Schmerz

*Die nächsten drei Fragen beziehen sich darauf, wie Ihre Schmerzen innerhalb der **letzten 24 Stunden** waren:*

3a. Bitte kreuzen Sie hier den **Schmerz** an, den Sie innerhalb der letzten 24 Stunden hatten, wenn Sie sich ruhig verhalten haben.

0	1	2	3	4	5	6	7	8	9	10

Kein Schmerz — Stärkster vorstellbarer Schmerz

3b Wie stark waren Ihre Schmerzen innerhalb der letzten 24 Stunden, wenn Sie sich belastet haben, zum Beispiel bei **Mobilisierung, Bewegen, Waschen, Husten, Durchatmen?**

0	1	2	3	4	5	6	7	8	9	10

Kein Schmerz — Stärkster vorstellbarer Schmerz

3c. Bitte kreuzen Sie hier den **stärksten Schmerz** an, den Sie innerhalb der letzten 24h hatten.

0	1	2	3	4	5	6	7	8	9	10

Kein Schmerz — Stärkster vorstellbarer Schmerz

*Die nächsten vier Fragen beziehen sich darauf, ob bestimmte Tätigkeiten oder Ihre Stimmung innerhalb der letzten 24 Stunden **durch den Schmerz beeinträchtigt** waren. Mit „beeinträchtigt“ ist gemeint: die Tätigkeit ist **unmöglich** oder **nur unter großer Mühe möglich**. Waren Sind Sie durch die Schmerzen beeinträchtigt:*

4a. bei **Bewegung?**

❑ Ja ❑ Nein

4b. beim **Husten oder tiefen Luftholen?**

❑ Ja ❑ Nein

4c. beim **Schlafen?**

❑ Ja ❑ Nein

4d. in Ihrer **Stimmung?**

❑ Ja ❑ Nein

Copyright Winfried Meißner/ Uniklinikum Jena. Nutzung durch Dritte vorbehaltlich Genehmigung.

Die nächsten 4 Fragen beziehen sich auf Ihren ***Allgemeinzustand*** in den letzten 24 Stunden:

5a. Haben Sie sich sehr **müde** gefühlt?

❑ Ja ❑ Nein

5b. Haben Sie unter **Übelkeit** gelitten?

❑ Ja ❑ Nein

5c. Haben Sie unter **Schwindel** gelitten?

❑ Ja ❑ Nein

5d. Leiden Sie derzeit unter Verstopfung?

❑ Ja ❑ Nein

6. Wurden Sie über die verschiedenen **Möglichkeiten Ihrer Schmerztherapie** informiert?

❑ Ja ❑ Nein

7. Hätten Sie gewünscht, **mehr Mittel gegen Schmerzen** zu bekommen?

❑ Ja ❑ Nein

8. Wenn Sie Schmerzen während Ihres Aufenthaltes im Krankenhaus hatten bzw. Schmerztherapie erhalten haben (z.B. Medikamente, Schmerzsalben, Schmerzkatheter, Krankengymnastik, Wärme, Kälte, Gespräch usw.) kreuzen Sie bitte an, wie **zufrieden** Sie mit dem Ergebnis der Behandlung Ihrer Schmerzen sind.

**0**	**1**	**2**	**3**	**4**	**5**	**6**	**7**	**8**	**9**	**10**

Völlig unzufrieden — Sehr zufrieden

❑ Ich hatte keine Schmerzursache/nie Schmerzen und habe auch keine Schmerzbehandlung erhalten.

Vom Befragenden auszufüllen: Patient wurde interviewt: ❑ Ja ❑ Nein

Copyright Winfried Meißner/ Uniklinikum Jena. Nutzung durch Dritte vorbehaltlich Genehmigung.
